# Pro-oxidant antioxidant balance in patients with non-alcoholic fatty liver disease 

**Published:** 2019

**Authors:** Bibi Fatemeh Nobakht Motlagh Ghoochani, Mohsen Ghafourpour, Fateme Abdollahi, Shima Tavallaie

**Affiliations:** 1 *Noncommunicable Diseases Research Center, Neyshabur University of Medical Sciences, Neyshabur, Iran*; 2 *Department of Basic Medical Sciences, Neyshabur University of Medical Sciences, Neyshabur, Iran*; 3 22 *Bahman hospital, Neyshabur University of Medical Sciences, Neyshabur, Iran*; 4 *Department of Biochemistry, Faculty of Medicine, Mashhad University of Medical Sciences, Mashhad, Iran *

**Keywords:** Oxidative stress, NAFLD, Pro-oxidants/ antioxidants balance

## Abstract

**Aim:**

We aimed to determine these parameters in patients with non-alcoholic fatty liver disease using pro-oxidant antioxidant balance assay.

**Background:**

In human, pro-oxidants and antioxidants are normally produced and there is a balance between production and deletion of them. When the balance between oxidants and antioxidants are disrupted oxidative stress occurs. Oxidative stress is known one of the main mechanisms for the development of nonalcoholic fatty liver disease. Many investigations have evaluated some oxidants and/or antioxidant status in these patients. However, studies explaining the antioxidant status and the oxidant burden in these patients are lacking.

**Methods:**

Sera from 35 healthy subjects and 38 patients with non-alcoholic fatty liver disease were recruited. Then, the pro-oxidant burden and the antioxidants capacity were measured by pro-oxidant antioxidant balance assay.

**Results:**

There was no significant difference in the mean pro-oxidant antioxidant balance values between the two study groups. The results demonstrated that serum pro-oxidant antioxidant balance values were positively correlated with BMI and age in the patient group. Furthermore, the pro-oxidant antioxidant balance significantly increased in women when compared with men in all participants.

**Conclusion:**

It demonstrated that increased antioxidant status could be as a response reflecting of the organism to elevated oxidants in NAFLD patients which may lead to unchanged PAB values.

## Introduction

 Non-alcoholic fatty liver disease (NAFLD) is the most common liver disorder in the population worldwide, its incidence is rising with obesity and diabetes mellitus because of alterations in lifestyle and diet. The prevalence of NAFLD among adults and children in developed countries is approximately 30 and 10%, respectively (1). 

Accumulation of fats in the hepatocytes has been linked to the pathogenesis of NAFLD. Subsequently, oxidation of fat increases in order to prevent fat accumulation and can lead to elevated levels of oxidative stress (2). Oxidative stress is one the most important causes of progression of NAFLD (3). Many studies demonstrated high levels of reactive oxygen species (ROS) and lipid peroxidation products in these patients (3-5). However, investigations explaining the antioxidant status in patients with NAFLD are conflicting. Some of these studies reported significant decreases in antioxidant levels in NAFLD patients in comparison with healthy controls (3), whilst others showed unchanged and increased levels of these markers (6, 7). Of note, in previous studies, one or several oxidant(s) and/or antioxidant(s) have been measured separately as a result that these measurements cannot be generalized to total oxidants and antioxidants status. Therefore, further studies and novel approaches are required to measure the pro-oxidant burden and the antioxidants capacity in these patients. 

Alamdari *et al.* (8) developed an approach named pro-oxidants/ antioxidants balance (PAB) in which the ratio of pro-oxidants to antioxidants are reported in one assay using 3,30,5,50-tetramethylbenzidine (TMB), this ratio presents redox index. 

Because of controversial reports about antioxidant levels in NAFLD patients that mentioned above, this study was conducted to assess PAB in these patients and its correlation with biochemical and anthropometrical parameters. This is the first time that the PAB assay is measured in NAFLD patients. 

## Methods

**Chemicals**

TMB powder (3,30,5,50-tetramethylbenzidine 2 HCl) is purchased from Sigma (Helsinki, Finland), peroxidase enzyme is purchased from Applichem (230 U/mg, A3791, 0005, Darmstadt, Germany), chloramine T trihydrate is purchased from Applichem (A4331), hydrogen peroxide (30%) and other materials such as acetic acid (HCl), sodium hydroxide (NaOH) and uric acid were purchased from Merck. 

**Study population**

A total of 73 participants were recruited for this study from 22 Bahman Hospital (Neyshabur, Iran) between January and August 2017. Of these 38 (ages 25-62) were diagnosed as NAFLD patients and 35 (ages 23-65) were healthy subjects. Of 38 NAFLD patients, 8 patients had hyperlipidemia and 3 patients had high blood pressure; therefore, these subjects received medications associated with their disorders. All patients met following inclusion criteria: (1) diagnosis of NAFLD using clinical and hepatic sonography (2) having 20-65 years of age , (3) being non-smoker and non-alcoholic (less than 20 mg/day), (4) non-pregnant and non-lactating women and exclusion criteria were included: (1) subjects with other disorders of acute and chronic liver such as viral and autoimmune hepatitis, hemochromatosis, Wilson’s disease, and alcoholic liver disease, (2) history of medical disorders, including diabetes mellitus, hyper/hypothyroidism, cancer, cardiovascular diseases. 

To confirm absence of NAFLD in the control group, hepatic ultrasonography was carried out for all healthy volunteers. Also, controls were matched for age and gender with patients. The protocols of this study were approved the Ethics Committee of Neyshabur University of Medical Sciences (NUMS). Written informed consent was obtained from each subject included in this work.

**Anthropometric and biochemical parameters**

For each subject, anthropometric measurements were taken using weight, height, and body mass index (BMI). BMI was calculated using the formula BMI = Weight/(Height)^2^. Furthermore, blood sample was drawn from each subject after an overnight fasting period. Blood samples were centrifuged at 3000 rpm for 10 min and serum was separated and biochemical measurements were performed immediately after sampling by clinical routine methods. Residual serum stored in aliquots at -80 ^º^C until PAB analyses were performed.

**Table 1:** Anthropometric and clinical characteristics of participants

	Control (n=35)	NAFLD (n=38)	*p*-value
Gender (M/F)	13/22	17/21	0.635
Height (cm)	164.6±1.52	163.77±1.96	0.740
Weight (kg)	66.23±1.76	79.00±2.03	0.000
BMI (kg/m^2^)	24.43±0.57	29.63±0.81	0.000
FBS (mg/dl)	90.05±1.15	92.29±1.99	0.336
TC (mg/dl)	174.47±5.64	195.37±7.22	0.026
TG (mg/dl)	106.67±8.74	147.1±11.91	0.008
LDL-C (mg/dl)	103.20±4.28	105.19±4.79	0.758
HDL-C (mg/dl)	47.00±1.21	45.40±1.68	0.446
ALT (IU/L)	21.67 (8-48)	41.00 (13-157)	0.000
AST (IU/L)	18.11±1.01	28.43±2.70	0.001
ALP (U/L)	163.58±7.54	193.13±9.17	0.015
T Bili (mg/dl)	0.80±0.04	0.83±0.06	0.663
D Bili (mg/dl)	0.21 (0.1-0.4)	0.24 (0.07-0.41)	0.264
PAB (HK)	97.47±5.7	98.90±6.71	0.871

Values are presented as mean±SE (parametric analysis) and mean (range) (non-parametric analysis). FBS: Fasting blood sugar; TC: Total cholesterol; TG: Triglyceride; LDL: Low-density lipoprotein cholesterol; HDL: High-density lipoprotein cholesterol; ALT: Alanine aminotransferase; AST: Aspartate aminotransferase; ALP: Alkaline phosphatase; T Bili: Total bilirubin; D Bili: Direct bilirubin. Comparison between two groups (control *vs*. NAFLD patients) using Fisher's exact test (for categorical variables such as gender), the independent t-test (for normal distributed data: all variables except for gender, ALT and D Bili), and Mann-Whitney U test (for non-normal distributed data: ALT and D Bili).

**Figure 1:**
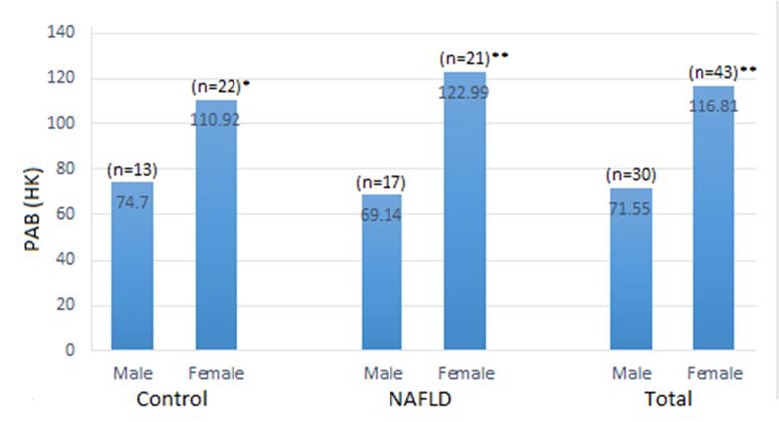
Change in pro-oxidant–antioxidant balance (PAB) based on gender between the study groups. PAB data were expressed as mean. There was a significant increase in PAB values in females between the study groups. *p < 0.05, female versus male in the control group; *p < 0.001, female versus male in NAFLD and total participants

**PAB assay**

PAB assays were performed according to our previous studies (9, 10). It is included two different types of reactions: an enzymatic reaction in which pro-oxidants oxidize the chromogen TMB to a color cation and the second reaction, a chemical reaction, in which antioxidants reduce the TMB cation to a colorless compound (8). 

For PAB assay, three solutions were prepared (TMB cation solution, TMB solution, and working solution). First, 60 mg of the TMB powder was dissolved in DMSO (10 mL). The TMB cation solution was prepared by adding the TMB/DMSO solution (400 mL) to acetate buffer (20 mL, 0.05M buffer, pH 4.5), and then inserting fresh chloramine T (70 mL, 100 mM). The obtained solution was incubated for 2 hours at room temperature in a dark place; subsequently, 25 U of peroxidase enzyme solution was inserted. This mixture stored in small (1 mL) aliquots at -20 ^º^C. For preparation of TMB solution, TMB/DMSO (200  L) was added to acetate buffer (10 mL, 0.05 M buffer, pH 5.8). The working solution was prepared by inserting TMB cation solution (1 mL) to TMB solution (10 mL). This solution was incubated for 2 min at room temperature in a dark place and immediately used. Furthermore, for preparation of the standard solutions, different proportions (0–100%) of hydrogen peroxide (250 mM) were mixed with uric acid (3 mM) in NaOH (10 mM).

In each well of a 96-well plate, 200  L of working solution was inserted into 10  L of each sample, standard, or blank (distilled water), which was incubated in a dark place at 37^o^C. After 12 min incubation, HCl (100  L, 2 N) was added to every well, then the optical density (OD) was evaluated using an ELISA reader (Stat Fax-2100, USA) at 450 nm, with a reference wavelength of 620 or 570 nm. The standard samples are used to plot the standard curve. To calculate the PAB values of the unknown samples, the standard curve was used. The PAB values may be expressed as arbitrary units (HK), according to percentage of hydrogen peroxide in the standard solution. 

**Statistical analysis**

Statistical analysis was performed using SPSS (version 16 software; SPSS Inc., Chicago, IL). A *p* value <0.05 with a confidence interval of 95% was considered statistically significant. To assess normality distribution of the data in each of the research arms, the Shapiro–Wilk test was used. Continuous variables are expressed as mean±SE (for variables with a normal distribution) and mean (range) (for variables with a non-normal distribution). Fisher's exact test was used for categorical variables such as gender. Participant characteristics between the two groups (the patients and the controls) were compared using the independent t-test (for normally distributed data) and Mann-Whitney U test (for non-normal distributed data) for continuous variables. Bivariate correlations between different parameters and PAB values were performed using Pearson correlation coefficient and Spearman's rank correlation for normal and non-normal distribution data, respectively. Analysis of covariance (ANCOVA) was used to adjust confounding factors such as BMI and age between the two study groups. 

## Results

**Anthropometric characteristics and biochemical analysis**

Anthropometric measurements and biochemical analysis of the study groups are summarized in Table 1. There were significant differences between the study groups regarding weight and BMI. 

As shown in Table 1, biochemical analysis revealed that patient group had significantly higher triglyceride (TG), total cholesterol (TC) and liver enzymes (aspartate aminotransferase (*AST*), alanine aminotransferase (*ALT*), and *alkaline phosphatase (ALP)*) values compared to the control group. Other biochemical parameters were in the normal range between the two groups. 

**PAB values between the control and NAFLD patients**

Mean PAB value in healthy volunteers was 97.47±5.7 HK, which was not significantly different from patient group (98.90±6.71, p>0.05) (Table 1).

**Association** **between PAB values and anthropometric and biochemical parameters**

The results demonstrated that serum PAB values were positively correlated with BMI (r= 0.544, p= 0.001) and age (r= 0.684, p< 0.001) in NAFLD group. Furthermore, there was also positive correlation between BMI and age (r= 0.55, p= 0.001) in NAFLD patients.

Sub-group analysis was performed based on gender. The results indicated that PAB values significantly decreased in male subjects as compared with female participants in all groups (Figure 1).

## Discussion

To the best our knowledge, this is the first time that the PAB assay is measured in NAFLD patients. Since previous works measured one or several oxidant(s) and/or antioxidant(s) separately, these evaluations could not represent oxidative stress (OS). In this regard, PAB seems to be a better method than individual markers due to in the body there is a strong interaction between oxidants and antioxidant and what is evaluated by PAB is the ratio of pro-oxidants to antioxidant simultaneously.

No significant difference was observed in PAB values between NAFLD patients and the control group (*p*-value = 0.87), even after adjusting by confounding factors (BMI and age: *p*-value = 0.12) with ANCOVA. This is in line with prior report that showed lipid peroxidation products, as oxidants, and total antioxidant capacity (TAC) increased in NAFLD patients when compared to healthy controls (7). Furthermore, Perlemuter *et al.* measured the activities of some antioxidant enzymes including cytosolic and mitochondrial superoxide dismutases (Cu/Zn-SOD and Mn-SOD), glutathione peroxidase and catalase as well as the levels of lipid peroxidation products in liver tissue and erythrocytes from NAFLD patients in comparison with the control group (11). They showed that there were no significant differences in the activities of erythrocyte antioxidant enzymes and lipid peroxidation products in two groups, while the activities of liver antioxidant enzymes, except for Mn-SOD activities and lipid peroxidation products levels, elevated in NAFLD patients (11). Likewise, Malaguarnera *et al.* demonstrated that heme oxygenase 1, an antioxidant enzyme, was induced in NAFLD patients (12). Another study reported that mRNA and protein expression of some antioxidant enzymes including NAD(P)H:quinone oxidoreductase 1 (NQO1), glutathione transferase (GST), and glutamate cysteine ligase as well as their isoforms altered in human NAFLD progression (13). For instance, mRNA and protein levels and activity of NQO1 increased with disease progression. mRNA levels of GST isoforms A, M, and P enhanced with disease progression. Additionally, protein levels of these isoforms increased except for GST M which attenuated. Their results indicated that the levels of mRNA and proteins of enzymes involved in the antioxidant response increased or unchanged in NAFLD patients (13). 

Besides, oxidant markers such as 4-hydroxynonenal (HNE) (14) and lipid peroxidation products (7, 12, 14, 15) were increased in patients with NAFLD and liver failures.

According to our findings and previous studies, we suggested that pro-oxidants increased in NAFLD patients as a result an adaptive response against oxidative damage induced by antioxidants/antioxidant enzymes. Increases in uric acid (16, 17), as an antioxidant, and nuclear factor E2-related factor 2 (Nrf2) (13, 18), a regulator of antioxidant defense, that were observed in NAFLD patients might confirm this claim. 

In this study, the findings from the coefficient of correlation in NAFLD group suggested that PAB values were significantly related to the BMI. This result is in agreement with prior investigations that reported adipose tissue is the main source of the increased plasma ROS (19). In obese subjects, elevated OS is mediated through elevated expression of NADPH oxidase subunits, decreased mRNA expression of antioxidant enzymes and adiponectin as well as increased levels of inflammatory markers such as leptin (19-22). Decreased adiponectin and elevated leptin, released hormones by the adipose tissue, have been shown in the circulation in obese subjects (20, 21, 23). In humans, serum adiponectin has been negatively correlated with systemic OS (22, 24). Serum leptin stimulates other inflammatory markers such as C-protein reactive (CRP) in hepatocytes (20, 21). Since inflammation and OS are related to one another; therefore, leptin is positively correlated with OS in obese population (25, 26). 

Another finding from the coefficient of correlation in NAFLD patients indicated that PAB values were positively correlated with age. Besides, there was also positive correlation between BMI and age (r= 0.55, *p*= 0.001) in NAFLD patients. As reported previously, the aging is one of the major causes of reduction in the antioxidant capacity (27, 28); however, because controls were matched for age and gender with patients as a result increases in PAB values in older subjects could be due to elevated BMI. 

Interestingly, our results showed that women in this study had higher values of PAB than men in two other study groups. We assumed that the higher BMI in women may be a factor, but adjusting the data for BMI did not change the sex effect. There are conflicting reports regarding the relation of gender to OS. Several authors show increased evidence of OS in men as compared with women (29, 30), while others demonstrated decreased levels of OS (31, 32). Of note, the previous studies did not measure total oxidants and antioxidants, but this study is interesting to evaluate the ratio of pro-oxidants to antioxidants. However, further studies with larger population are required to confirm this finding.

There are several limitations that can affect our results. First, we collected wide range of age in both groups of participants. Second, we did not evaluate some confounding factors including diet patterns and drug usages. Third, participants of this study were from small region of Iran. Given that racial and ethnic differences may influence NAFLD prevalence and severity, in future studies are recommended to collect larger community in order to confirm our findings.

Our results indicated that no significant difference was observed in PAB values between NAFLD patients and the healthy controls. This finding might suggest that enhanced antioxidant capacity could be as a response reflecting of the organism to increased oxidants in NAFLD patients, resulting in unchanged PAB values. However, further investigations with larger population are needed to confirm these results. Furthermore, this study suggested that increases in BMI may contribute to changed oxidative status in patients with NAFLD. Another finding is that women had significantly higher PAB values than men, even after adjustment for BMI
